# Molecular detection and genotyping of bovine viral diarrhea virus in Western China

**DOI:** 10.1186/s12917-021-02747-7

**Published:** 2021-02-02

**Authors:** Lingling Chang, Yanping Qi, Dan Liu, Qian Du, Xiaomin Zhao, Dewen Tong

**Affiliations:** College of Veterinary Medicine, Northwest A&F University, Yangling, Shaanxi China

**Keywords:** BVDV, RT-PCR, Genotype, Bovine, Western China

## Abstract

**Background:**

Bovine viral diarrhea virus (BVDV) is an important global viral pathogen of cattle and other ruminants. To survey the infection rate and genetic diversity of BVDV in western China, a total of 1234 serum samples from 17 herds of dairy cattle, beef cattle and yak in 4 provinces were collected in 2019.

**Results:**

All the 1234 serum samples were screened individually for BVDV by RT-PCR. Our results demonstrated that the average positive rate of BVDV was 7.2% (89/1234) in animals and 82.4% (14/17) in herds. Thirteen BVDV strains were isolated from RT-PCR positive clinical samples and they were all NCP biotype. BVDV-1a and 1c subgenotypes were identified from 22 selected virus isolates in 14 BVDV-positive herds. These results confirmed that BVDV-1a and BVDV-1c were circulating in western China, similar to the BVDV epidemics in cattle in other regions of China.

**Conclusions:**

This study provides data for monitoring and vaccination strategies of BVDV in western China.

**Supplementary Information:**

The online version contains supplementary material available at 10.1186/s12917-021-02747-7.

## Background

Bovine viral diarrhea virus (BVDV) is an important pathogen of cattle worldwide and causes significant economic losses. BVDV has been detected in not only in cattle, but also in diverse domestic [1, 2] and wildlife animal species [3–5].

BVDV is a member of the *Pestivirus* genus within the family *Flaviviridae*. There are two common BVDV genetic species, BVDV-1 and BVDV-2. However, a newly recognized pestivirus species, “HoBi-like” or “atypical pestiviruses” has been considered as the third genetic species of BVDV [6, 7], and entitled as BVDV-3.The BVDV genome consists of a single-stranded, plus-sense RNA approximately 12.3 kb in length, which is flanked by 5′and 3′untranslated regions (5’UTR, 3’UTR) and encodes 11–12 structural and non-structural proteins (Npro, C, Erns, E1, E2, P7, NS2/3, NS4A, NS4B, NS5A, NS4B). BVDV can be divided into two biotypes, cytopathic (CP) and noncytopathic (NCP), based on its ability of the production of the visible effects on cell culture [8]. The 5’UTR region has primarily been used for subgenotype identification as well as N^pro^ and E2 regions [7, 9–15]. On the basis of the 5’UTR, various subgenotypes of BVDV isolates have been identified. To date, 21 subgenotypes within the BVDV1 (1a-1u) and 3 subgenotypes of BVDV2 have been reported worldwide [10, 14, 16], which predominate in different countries. In China, nine subgenotypes have been identified in cattle, including 1a, 1b, 1c, 1d, 1 m, 1o, 1p, 1q, and 1u [17, 18].

Previous studies showed that a high proportion of BVDV-positive cattle came from western China [19, 20], because these areas are historically the main regions with cattle production. However, studies regarding the genetic diversity of BVDV in western China remain rare [4, 21, 22]. Thus, this study detected and genotyped BVDV from bovines in this region. Such studies are important to understand the diversity of viral strains present in one region and this, in turn, can inform control programs, drive vaccine development and determine likely infection sources.

## Results

### Detection of BVDV in clinical serum samples

A total of 1234 serum samples were tested for BVDV by 5’UTR RT-PCR. As shown in Table 1, the average positive rate of BVDV in animals was 7.2% (89/1234). At the herd level, a herd was considered positive if one serum sample was positive in RT-PCR testing. Thus, 82.4% (14/17) of herds were positive and they were located in Shaanxi, Ningxia, Xinjiang, and Tibet. No BVDV infection was found in 3 herds from Shaanxi and Ningxia Provinces. The average positive rate of BVDV in Shaanxi, Ningxia, Xinjiang, and Tibet was 4.57% (44/963), 30% (18/60), 37.5% (6/16) and 10.77% (21/195), respectively.

**Table 1:** Samples collected and RT-PCR detection of BVDV

Herd No.	Provinces	Species	Date	Clinical symptoms	No. sample collected	No. positive sample	Positive rate in sample	Status in herd	Positive rate in province
1	Shaanxi	Dairy	2019.3	Diarrhea	45	0	0(0/45)	–	4.57%(44/963)
2		Beef	2019.4	Diarrhea	47	0	0(0/47)	–	
3		Diary	2019.5	Healthy	137	5	3.65%(5/137)	+	
4		Diary	2019.5	Diarrhea	29	2	6.90%(2/29)	+	
5		Beef	2019.5	Healthy	211	10	4.74%(10/211)	+	
6		Diary	2019.5	Diarrhea	43	8	18.60%(8/43)	+	
7		Beef	2019.6	Healthy	116	9	7.76%(9/116)	+	
8		Beef	2019.6	Diarrhea	25	1	4%(1/25)	+	
9		Diary	2019.12	Diarrhea	280	7	2.50%(7/280)	+	
10		Diary	2019.12	Diarrhea	30	2	6.67%(2/30)	+	
11	Ningxia	Diary	2019.4	Diarrhea	13	2	15.38%(2/13)	+	30%(18/60)
12		Diary	2019.4	Diarrhea	10	6	60%(6/10)	+	
13		Beef	2019.4	Diarrhea	15	10	66.67%(10/15)	+	
14		Diary	2019.9	Diarrhea	22	0	0(0/22)	–	
15	Xinjiang	Diary	2019.8	Diarrhea	16	6	37.50%(6/16)	+	37.5%(6/16)
16	Tibet	Yak	2019.7	Diarrhea	98	15	15.31%(15/98)	+	10.77%(21/195)
17		Yak	2019.9	Diarrhea	97	6	6.19%(6/97)	+	
					1234	89	7.2%(89/1234)	^a^82.4%(14/17)	

^**a**^ Rate of positive herds among all the tested herds

### Virus isolation

BVDV strains were isolated from RT-PCR positive clinical samples and they were identified by immunofluorescence, RT-PCR and sequence analysis, and transmission electron microscopy.

**Fig. 1:**
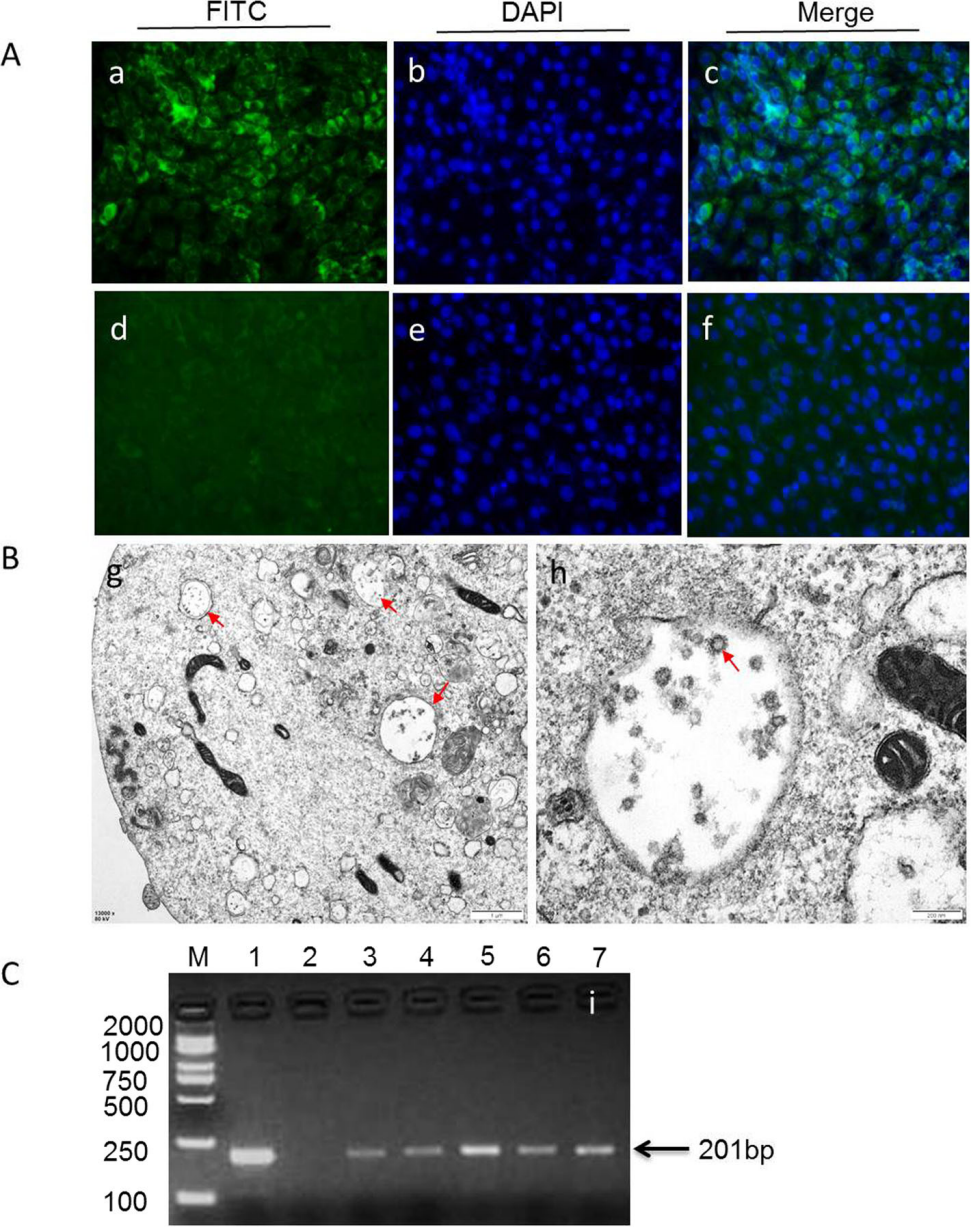
The isolated BVDV strains were confirmed by immunofluorescence, RT-PCR and transmission electron microscope, respectively. **a** BVDV-infected (**a**-**c**) or negative control MDBK cells (**d**-**f**) were examined by immunofluorescence using polyclonal antibodies against BVDV E2 protein. **b** The typical viral particles in the cytoplasm of infected MDBK cell(g, red arrow). The viral particles were measured approximately 60 nm in diameter and occurred as clusters inside vesicles(h, red arrow). **c** The BVDV strains were detected by 5’UTR RT-PCR (201 bp). Representative electron microscopic image of field BVDV isolates (i). lane M:weight size marker (2000 bp,1000 bp, 750 bp, 500 bp, 250 bp,100 bp), lane 1: positive control; lane 2: negative control, lanes 3–7: BVDV strains isolated from clinical serum samples

Initially all of the 89 positive samples were subjected to virus isolation, but only 13 BVDV strains were successfully isolated (Fig. 1a-c; S1 Figure). The isolated BVDV strains were named as SX-01XN19, SX-02XN19, NX-05XN19, NX-69209, NX-03XN19, NX-59181, NX-59211, NX-04XN19, NX-10XN19, XJ-06XN19, XJ-07XN19, XJ-08XN19 and XZ-09XN19, respectively. All the 13 strains caused no obvious cell lesions, hence, they were identified as NCP biotype (Fig. 1a). Transmission electron microscopy examination showed typical viral particles in the cytoplasm of MDBK cell, which were measured approximately 60 nm in diameter and occurred as clusters inside vesicles (Fig. 1b). The information of these BVDV strains including source, biotype, and genotype was presented in Table 2.

**Table 2:** List of field virus isolates used in the study

Virus isolate	Herd	Material	Origin	Genotype	Biotype	GenBank accession no.
SX-02XN19	3	Cell culture	Shaanxi	1a	ncp	MT316318
SX-2	3	Serum	Shaanxi	1a	^a^-	MW142339
SX-1-2	4	Serum	Shaanxi	1a	–	MW142341
SX-01XN19	5	Cell culture	Shaanxi	1c	ncp	MT316327
SX-3	6	Serum	Shaanxi	1a	–	MW142340
SX-219	7	Serum	Shaanxi	1a	–	MW142338
SX-1-1	8	Serum	Shaanxi	1a	–	MW142343
SX-3-46	9	Serum	Shaanxi	1a	–	MW142342
SX-3-2	9	Serum	Shaanxi	1a	–	MW142344
SX-3-53	10	Serum	Shaanxi	1a	–	MW142345
NX-69209	11	Cell culture	Ningxia	1a	ncp	MW142337
NX-03XN19	12	Cell culture	Ningxia	1c	ncp	MT316319
NX-59181	12	Cell culture	Ningxia	1c	ncp	MW142336
NX-59211	12	Cell culture	Ningxia	1c	ncp	MW142335
NX-04XN19	12	Cell culture	Ningxia	1c	ncp	MT316320
NX-10XN19	12	Cell culture	Ningxia	1c	ncp	MT316325
NX-05XN19	13	Cell culture	Ningxia	1a	ncp	MT316321
XJ-06XN19	15	Cell culture	Xinjiang	1a	ncp	MT316322
XJ-07XN19	15	Cell culture	Xinjiang	1a	ncp	MT316323
XJ-08XN19	15	Cell culture	Xinjiang	1a	ncp	MT316324
XZ-N24	16	Serum	Tibet	1a	–	MW142346
XZ-09XN19	17	Cell culture	Tibet	1c C	ncp	MT316328

^a^The sample was not successfully isolated from cell culture

### Sequencing and phylogenetic analysis

To investigate the extent of genetic diversity of BVDV in western China, the subgenotypes of the BVDV isolates were determined by 5’UTR sequencing and phylogenetic analysis.

**Fig. 2:**
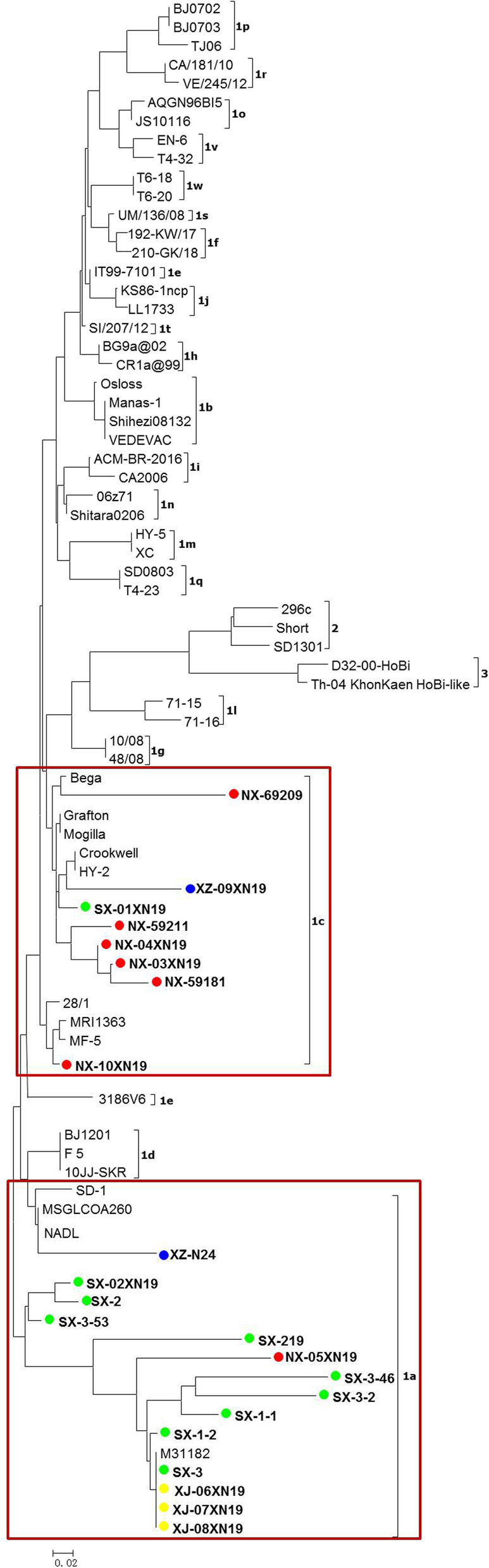
Phylogenetic analysis based on 5’UTR sequence. A phylogenetic tree of the 5’UTR was created using the 5’UTR sequences of 22 selected BVDV isolates and 57 reference isolates retrieved from the GenBank database. ●BVDV isolates identified in provinces of Shaanxi (green dot), Ningxia (red dot), Xinjiang (yellow dot) and Tibet (blue dot) in this study.14 isolates were clustered in BVDV-1a (frame) and 8 isolates clustered in BVDV-1c (frame)

As shown in Tables 2, 22 virus isolates, including 13 isolated strains and 9 positive serum samples, were randomly selected from 14 BVDV positive herds and used for phylogenetic analysis. BLAST search revealed that all sequences belonged to BVDV-1. As shown in Fig. 2, phylogenetic analysis revealed that these isolates clustered into either BVDV-1a (*n* = 14) or BVDV-1c (*n* = 8) subgenotypes. The BVDV-1a subgenotypes were located in Shaanxi (*n* = 9), Ningxia (*n* = 1), Xinjiang (*n* = 3) and Tibet (*n* = 1), and the BVDV-1c located in Shaanxi (*n* = 1), Ningxia (*n* = 6) and Tibet (*n* = 1).

## Discussion

In this study, we investigated the prevalence and genetic diversity of BVDV among bovines in western China. The BVDV positive rate varies greatly among bovine herds due to the variety of detection tests, sampling methods, species, and locations. Hence, it is difficult to determine the exact extent of the BVDV prevalence in China. As previously reported, RT-PCR analysis of BVDV RNA is more sensitive than other Ag detection methods and has been widely used for BVDV detection. Primer is pivotal for the accuracy and sensitive of RT-PCR detection. In this study, the primers BP189–389 for 5’UTR region was used, which had a broad range of bovine pestivirus detection including BVDV-1, BVDV-2 and BVDV-3 [23].

Our results demonstrated that the average positive rate of BVDV in animals was 7.2% (89/1234). Previous reports showed that the RNA prevalence of BVDV was 22.64% among bovine in China [17]. A systematic review and meta-analysis showed that the RNA prevalence of BVDV was 27.1% in dairy cattle in China [19]. In our study, there were limitations in the samplings and detection methods. Most of the clinical samples were sent to our laboratory by local farmers without providing detail information on the farms such as size and position. In addition, the sampling site did not completely cover the entire regions in western China. Considering the experimental expenses, the serology test was not performed in this study. Hence, the average RT-PCR positive rate of 7.2% in animals may not reflect the accurate prevalence of BVDV among bovines in western China. Notably, however, at herd level, we found 82.4% (14/17) of herds were positive for BVDV. Recently, a high prevalence of 57.78% of herds was reported to be positive for BVDV in northwestern China [1]. Our results suggested that a high proportion of herds are at risk of BVDV infection in western China.

Virus isolation is the standard method of detection of BVDV-infected cattle. The BVDV infected animals can secrete large amounts of BVDV in their serum, especially persistent infection (PI) animals. Previous studies demonstrated that BVDV remained viable in serum under normal conditions of sample submission to a diagnostic laboratory. Hence, the serum is a valid sample for the isolation of BVDV. In this study, 13 noncytopathic (NCP) strains were successfully isolated from clinical serum samples. NCP biotype is commonly found in nature. Our results further confirmed those of other workers.

BVDV is an RNA virus with high mutation rate [24]. Study investigating the frequency and number of subgenotypes of BVDV is helpful to understand the evolution of the virus and the source of infection. This information also has important implications for the design and construction of effective vaccination strategies to control BVDV [25, 26]. In this study, two subgenotypes of BVDV1a and BVDV1c were identified among 22 selected virus isolates from 14 herds. These results were in agreement with recent epidemiologic studies of BVDV in cattles in China [20, 27]. BVDV2 and BVDV3 were not detected in this study.

Although the predominant subgenotype worldwide is BVDV-1b, BVDV-1a and -1c are the second and third most frequently-reported genotypes in the world, respectively [14].BVDV-1a is predominant in South Africa and is widely circulating in the United States, Korea and Japan [28], while BVDV-1c has been reported as a predominant genotype in Australia [22, 28]. In China, BVDV-1a and -1c have been commonly detected in different regions of China. Recently, BVDV-1c (7/9) and BVDV-1a (1/9) strains were detected from 36 herds of dairy cattle in 5 provinces in eastern China [29]. A recent analysis of 119 BVDV sequences obtained from 92 dairy farms showed that subgenotypes of BVDV-1a (*n* = 37, 31.09%), BVDV-1c (*n* = 34, 28.57%) and BVDV-1 m (*n* = 25, 21.01%) were predominant in 19 provinces of China in 2017 [20].

In western China, scattered studies on the genetic diversity of BVDV among cattle and other ruminants have been reported. Subgenotypes BVDV-1b and BVDV-1c have been identified in cattle from Xinjiang Autonomous Region [22]. BVDV-1b and BVDV-1d were found predominant subgenotypes in dairy farms in Ningxia Autonomous Region [21]. Six subgenotypes of BVDV-1a, BVDV-1b, BVDV-1c, BVDV-1 m, BVDV-1o, BVDV-1p and BVDV-1q have been identified in Bactrian camels from regions of Xinjiang, Gansu and Qinghai [4]. Here, BVDV-1a was respectively detected in Shaanxi, Ningxia, Tibet and Xinjiang, and BVDV-1c was detected in Shaanxi, Ningxia, and Tibet. Taken together, our results confirmed the presence of 1a and 1c subgenotypes in western China. The genetic diversity of virus isolates hamper prevention and control of BVDV. A vaccine effective in one region may fail to protect against virus infection caused by different virus strains in another region [30]. Our findings provide important information for further characterization of the variability and geographical distribution of BVDV in China.

## Conclusions

Our results demonstrated that the average positive rate of BVDV was 7.2% (89/1234) in animals and 82.4% (14/17) in herds. Thirteen BVDV strains were successfully isolated from RT-PCR positive clinical samples and they were all NCP biotype. BVDV-1a and 1c subgenotypes were identified from 22 selected isolates from 14 herds. These results confirmed that BVDV-1a and BVDV-1c were circulating in western China, similar to the BVDV epidemics in cattle in other regions of China. This study provides data for monitoring and vaccination strategies of BVDV in western China.

## Methods

### Clinical sample collection

A total of 1234 serum samples from 17 herds covering 4 provinces in western China (Shaanxi, Ningxia, Xinjiang, and Tibet) were collected in 2019. Most samples were collected from herds in which the animals showed diarrhea. Some samples were submitted from clinical healthy animals for conventional detection. The sample information is summarized in Table 1.These herds were not vaccinated against BVDV. The samples were stored at − 80 °C for RT-PCR and virus isolation.

### RT-PCR

The clinical serum samples or cell culture were examined for the presence of BVDV by RT-PCR. Briefly, total RNA was extracted from serum or cell culture using TRIzol Reagent (Gibco). cDNA was synthesized from 1000 ng of total RNA using RNA reverse transcription kit (invitrogen USA).

The synthesized cDNA were submitted sequentially to PCR assay to amplify a 201-bp fragment of the BVDV 5′-UTR region, using referenced primers BP189–389 [21] (Forward: 5′-AGTCGTCAATGGTTCGAC-3′; Reverse: 5′-TCCATGTGCCATGTACA-3′). All PCR reaction were performed in 15 μL volume containing 7.5 μL of 2 × PCR Master Mix (Qiagen), 2 μL of cDNA, 4.5 μL ddH_2_O, and 10 μM each of the primers. The reaction was carried out at 94 °C for 4 min, followed by 35 cycles of 94 °C for 30 s, 47 °C for 30 s, and 72 °C for 30 s, with a final elongation step of 72 °C for 7 min. The PCR products were checked by electrophoresis on 1% agarose gel.

### Virus isolation

To investigate the biotype of BVDV circulating in herds, BVDV strains were isolated from RT-PCR positive clinical samples using standard virus isolation techniques. Briefly, serum samples were placed on Madin-Darby bovine kidney (MDBK) cells for 1 h at 37 °C in a 5% CO2 atmosphere. The cells were washed twice with PBS and then DMEM with 2% fetal bovine serum (BVDV and BVDV antibody-free) was added and incubated for 4–5 days. Then the cultures were frozen and thawed three times and the clarified supernatant was passaged five times in MDBK cells. In the absence of cytopathic effect, the cells were fixed and stained by immunofluorescence as previously described [12]. The supernatants of the infected cells were further tested by RT-PCR described as above for the presence of BVDV nucleic acid.

### Transmission electron microscope

The MDBK cell culture infected by RT-PCR positive samples were examined for the presence of BVDV particles by transmission electron microscopy (TEM). Cells were fixed with 2.5% glutaraldehyde in sodium cacodylate buffer (0.2 M, pH 7.2), post-fixed with 1% buffered osmium tetroxide, dehydrated in acetone, and embedded in epoxy resin. The resin blocks were then cut into 60 nm thick ultrathin sections, stained with uranyl acetate and lead citrate, and observed in a TEM (TECNAI G2 SPIRIT BIO).

### Sequencing and phylogenetic analysis

The RT-PCR amplified fragments obtained from serum samples or cell culture were directly sequenced by Shanghai Sangon Biological Engineering Technology & Services Co., Ltd. (Shanghai, China). Multiple sequence alignment was performed using the ClustalW program in BioEdit software. Phylogenetic analysis of the 5’UTR region was performed with the neighbor-joining method in MEGA 7.0 software. In the phylogenetic tree, the evolutionary distances were computed using the Tamura 3-parameter model with 1000 bootstrap replicates.

A total of 57 reference sequences of known BVDV-1, BVDV-2 and BVDV-3 isolates were obtained from the NCBI GenBank database (S2 Table). The 22 5’UTR sequences obtained in this study were deposited in GenBank (accession numbers MT316318-MT316325; MT316327-MT316328; MW142335-MW142346), see Table 2.

## Supplementary Information

**Additional file 1.**

**Additional file 2.**

## Data Availability

All the data supporting the results in the current study is contained within the manuscript. Sequences from this study have been deposited in NCBI GenBank under accession numbers as followed: 13 5’UTR sequences from BVDV strains isolated from MDBK cell culture with accession no. MT316318-MT316325, MT316327-MT316328, MW142335-MW142337; 9 5’UTR sequences from clinical serum samples with accession no. MW142338-MW142346.

## Abbreviations

BVDV: Bovine viral diarrhea virus
ORF: Large open reading frame
RT-PCR: Reverse transcription polymerase chain reaction
PCR: Polymerase chain reaction
MDBK: Madin-darby bovine kidney cells
cDNA: Complementary DNA
CP: Cytopathogenic
NCP: Non-cytopathogenic
