# Supplementary material for: Molecular detection and genotyping of bovine viral diarrhea virus in Western China
Source: BMC Vet Res. 2021 Feb 2;17:66. doi: 10.1186/s12917-021-02747-7 (PMC7853163; doi:10.1186/s12917-021-02747-7)
Supplement: Supplementary file 1 — Additional file 1. [file 12917_2021_2747_MOESM1_ESM.docx]

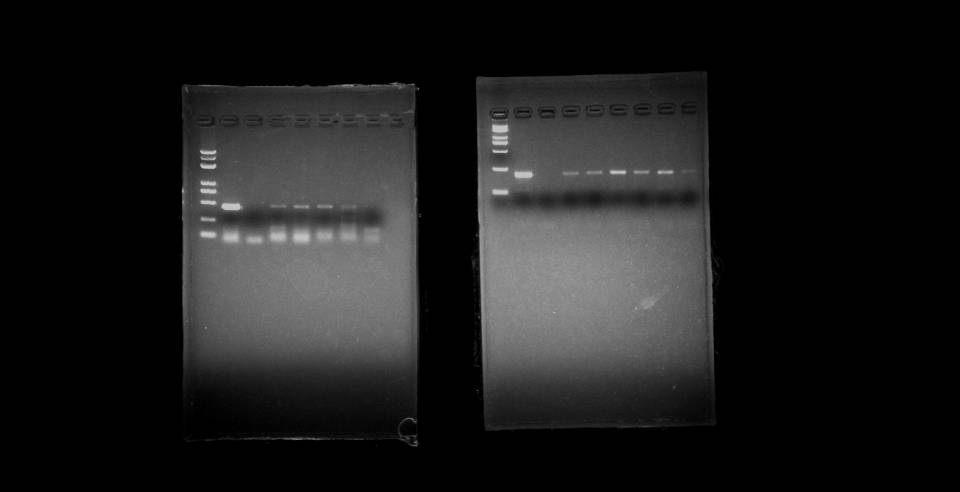


8

9

7

6

5

4

3

2

1

S1 Figure. The BVDV strains were detected by RT-PCR using primers for 5’UTR region (201 bp).

Figure legend: lane 1: weight size marker (2000 bp, 1000 bp, 750 bp, 500 bp, 250 bp, 100 bp), lane 2: positive control; lane 3: negative control, lanes 4-9: BVDV strains isolated from clinical serum samples.
