# Supplementary material for: Molecular detection and genotyping of bovine viral diarrhea virus in Western China
Source: BMC Vet Res. 2021 Feb 2;17:66. doi: 10.1186/s12917-021-02747-7 (PMC7853163; doi:10.1186/s12917-021-02747-7)
Supplement: Supplementary file 2 — Additional file 2. [file 12917_2021_2747_MOESM2_ESM.docx]

**S2 Table. Reference BVDV strains used in this study**

| BVDV strain | Genotype | GenBank | BVDV strain | | Genotype | GenBank |  |
| --- | --- | --- | --- | --- | --- | --- | --- |
| M31182 | 1a | M31182 | HY-5 | | 1m | KY865368 |  |
| SD-1 | 1a | M96751 | XC | | 1m | MH166806 |  |
| NADL | 1a | M31182 | 06z71 | | 1n | DQ973181 |  |
| MSGLCOA260 | 1a | MF347400 | Shitara0206 | | 1n | LC089876 |  |
| Manas-1 | 1b | EU555288 | AQGN96BI5 | | 1o | AB300691 |  |
| Osloss | 1b | M96687 | JS10116 | | 1o | JN248734 |  |
| Shihezi08132 | 1b | HQ015423 | BJ0702 | | 1p | GU120248 |  |
| VEDEVAC | 1b | AJ585412 | BJ0703 | | 1p | GU120249 |  |
| Bega | 1c | AF049221 | TJ06 | | 1p | GU120246 |  |
| Grafton | 1c | JQ743607 | SD0803 | | 1q | JN248727 |  |
| Mogilla | 1c | JQ743605 | T4-23 | | 1q | MN417855 |  |
| Crookwell | 1c | JQ743606 | CA/181/10 | | 1r | LM994672 |  |
| HY-2 | 1c | KY865365 | VE/245/12 | | 1r | LM994671 |  |
| MF-5 | 1c | KY865373 | UM/136/08 | | 1s | LM994673 |  |
| MRl1363 | 1c | LT901726 | SI/207/12 | | 1t | LM994674 |  |
| MF-5 | 1c | KY865373 | EN-6 | | 1v | MN417813 |  |
| BJ1201 | 1d | KT943518 | T4-32 | | 1v | MN417862 |  |
| F5 | 1d | AF298065 | T6-18 | | 1w | MN417892 |  |
| 10JJ-SKR | 1d | KC757383 | T6-20 | | 1w | MN417893 |  |
| 3186V6 | 1e | AF298062 | 296c | | 2a | MH806436 |  |
| IT99-7101 | 1e | AJ318618 | SD1301 | | 2b | KJ000672 |  |
| 192-KW-17 | 1f | MK381368 | Short | | 2c | MH231149 |  |
| 210-GK-18 | 1f | MK381386 | D32/00 HoBi | | 3 | AB871953 |  |
| 10/08 | 1g | JN715004 | Th-04 KhonKaen | | 3 | NC_012812 |  |
| 48/08 | 1g | JN715036 |  | |  |  |  |
| BG9a@02 | 1h | MG434576 |  | |  |  |  |
| CR1a@99 | 1h | MG434575 |  | |  |  |  |
| ACM/BR/2016 | 1i | KX857724 |  | |  |  |  |
| CA2006 | 1i | MK775204 |  | |  |  |  |
| KS86-1ncp | 1j | AB078950 |  | |  |  |  |
| LL1733 | 1j | GU987138 |  | |  |  |  |
| 71-15 | 1l | KF205306 |  | |  |  |  |
| 71-16 | 1l | KF205307 |  | |  |  |  |
|  |  |  | |  |  |  |  |
